# Prosthetic Valve Endocarditis Caused by *Pasteurella dagmatis*, Germany

**DOI:** 10.3201/eid3010.240727

**Published:** 2024-10

**Authors:** Felix A. Rottmann, Peter Schorle, Roland Giesen, Christoph Jäger

**Affiliations:** University of Freiburg Faculty of Medicine, Freiburg, Germany

**Keywords:** Pasteurella dagmatis, bacteria, prosthetic valve endocarditis, Germany, zoonoses, cats

## Abstract

An 81-year-old male patient in Germany had prosthetic valve endocarditis caused by *Pasteurella dagmatis* after a domestic cat bite. We surgically treated a paravalvular abscess and administered definitive antibiotic therapy consisting of penicillin G and levofloxacin. The patient was discharged from the intensive care unit in good condition 21 days after the surgery.

*Pasteurella* spp. are gram-negative, facultative anaerobic bacteria. They are part of the oral flora of domestic animals, including cats and dogs. In cases of animal bites, *Pasteurella* spp. bacteria can be detected culturally in 50%–75% of cases (*1*). Infections are typically caused by *Pasteurella multocida*, whereas *P. dagmatis* are rarely isolated from wounds, and severe *P. dagmatis* infections are exceedingly uncommon or unreported. Few cases of infective endocarditis (IE) attributable to *P. dagmatis* bacteria have been reported in the English-language literature (*2*–*5*). We report an additional case in a man in Germany. Given the rarity of gram-negative non-HACEK endocarditis (i.e., caused by species other than *Haemophilus* species, *Actinobacillus actinomycetemcomitans*, *Cardiobacterium hominis*, *Eikenella corrodens*, or *Kingella*) and the unfavorable prognosis associated with it, the contribution of case reports is essential to improve the management of affected patients (*6*).

An 81-year-old male patient was admitted to the hospital with a fever of up to 39.3°C. He reported weight loss of 10 kg during the preceding month. Vital signs and physical examination revealed no relevant pathologies. A domestic cat bite on the foot 2 months before admission was the only potential source of infection, but the wound had already healed weeks before. Underlying medical conditions included a mechanical aortic valve implanted 20 years previously because of a combined vitium.

After obtaining a set of blood cultures, we administered empiric antibiotic therapy with piperacillin/tazobactam. Results of chest radiograph, urine dipstick, and venous blood gas analyses were unremarkable. Results of point-of-care respiratory PCR testing for influenza and SARS-CoV-2 were negative. Blood testing showed mild anemia (hemoglobin 10.2 g/dL [reference range 11.6–15.5 g/dL]) but unremarkable leukocyte and platelet levels. C-reactive protein was moderately elevated at 47 mg/L (reference range <5 mg/L). Creatinine, urea, and liver function test results were unremarkable except for an international normalized ratio of 2.3 as a result of phenprocoumon therapy.

A set of blood cultures showed bacterial growth after 5 hours (anaerobic) and 10 hours (aerobic). We identified *P. dagmatis* bacteria by using matrix-assisted laser desorption/ionization time-of-flight mass spectrometry (MALDI Biotyper Sirius System; Bruker, https://www.bruker.com). The antibiogram demonstrated susceptibility to penicillin G, doxycycline, cotrimoxazole, and levofloxacin. Transthoracic echocardiography revealed a paravalvular abscess. We diagnosed prosthetic valve IE on the basis of modified Duke criteria (1 major clinical criterion, imaging; 3 minor clinical criteria, predisposition, fever, and microbiologic evidence falling short of a major criterion) (*7*).

The abscess measured at 24 ×14 × 31 mm on the subsequent computed tomography scan (Figure) and was communicating with the left ventricular outflow tract. The cardiac surgery department recommended abscess removal and surgical replacement of the mechanical valve and ascending aorta. The patient underwent paravalvular abscess resection and replacement of the prosthetic valve and ascending aorta with a 25-mm biological valve (Carpentier-Perimount Magna Ease; Edwards Lifesciences, https://www.edwards.com) and bovine pericardial patch plasty. Intraoperative molecular diagnostic tests (16S rRNA PCR) were positive for *P. dagmatis* bacteria.

**Figure:**
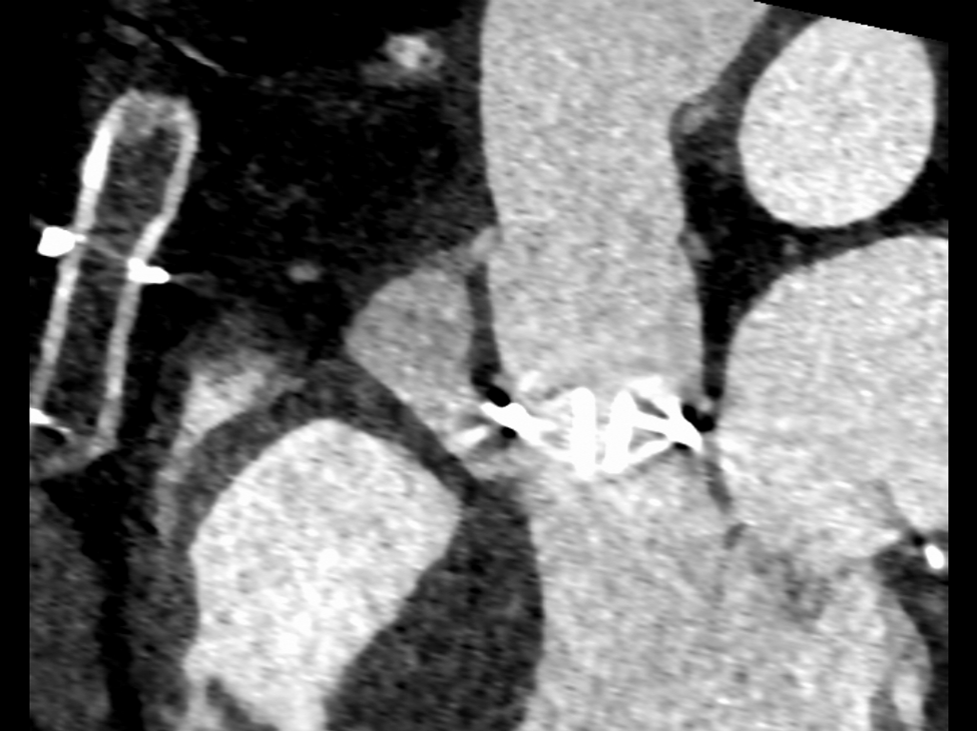
Preoperative contrast-enhanced computed tomography (sagittal) of an 81-year-old male patient in Germany who had *Pasteurella dagmatis* infection after a domestic cat bite, indicating a paravalvular abscess of 24 × 14 × 31 mm. The large abscess near his mechanical valve, combined with *P. dagmatis* bacteria in blood cultures, confirmed a diagnosis of infective endocarditis based on Duke criteria (*7*).

After surgery, the patient cardiogenic shock develop, requiring extracorporeal life support (ECLS). Because of bleeding attributable to ECLS, open chest management was necessary for 3 days. Acute kidney failure with anuria and hypervolemia (KDIGO acute kidney injury stage 3 [https://kdigo.org/guidelines/acute-kidney-injury]) resulted in dialysis for 5 days. ECLS could be explanted after 6 days. We treated sinus node dysfunction with a cardiac resynchronization therapy (pacemaker) device (Enitra 8 HF-T QP; Biotronik, https://www.biotronik.com) before intensive care unit discharge.

We continued to administer antibiogram-appropriate penicillin G therapy for a total of 6 weeks after the initial negative blood culture results. We administered levofloxacin, which we selected over an aminoglycoside antibiotic because of its superior abscess penetration, for the first 20 days as a combined therapy. The patient was discharged from the intensive care unit on the 21st day after surgery, exhibiting full consciousness and only mild focal neurologic deficits (dysphagia). He was discharged from the hospital shortly afterward and began rehabilitation treatment.

The choice of treatment in this case was complicated by the paucity of evidence regarding IE caused by *P. dagmatis* (*2*–*5*,*8*). Gump and Holden (*5*) reported IE caused by a new species of *Pasteurella* bacteria in 1972. In 1994, Sorbello et al. (*2*) reported another case, complicated by vertebral osteomyelitis. In 2001, Rosenbach et al. (*4*) reported prosthetic valve IE caused by *P. dagmatis*. A more recent case was reported in 2012 by Strahm et al. (*3*) (Appendix Table). Four of the 5 reported cases (including the case we describe) resulted from domestic cat bites, and all definitive therapies included either penicillin G or ceftriaxone. All patients survived infection. Antimicrobial therapy for *P. multocida* infection is similar to the course we describe, and penicillin has been recommended in all specimens that tested susceptible to it (*9*). 

Empiric antibiotic therapy for animal wounds varies by severity (ranging from amoxicillin/clavulanate to piperacillin/tazobactam) and also needs to target other potential pathogens. Cat bites can be easily underestimated and justify careful therapy and follow-up. Animal contact could have been missed in this case because the wound had healed fully before the first symptoms of IE. Obtaining the patient’s history greatly helped in the diagnosis.

Appendix Additional information about prosthetic valve endocarditis caused by *Pasteurella dagmatis*, Germany.
