# Supplementary material for: Prosthetic Valve Endocarditis Caused by Pasteurella dagmatis, Germany
Source: Emerg Infect Dis. 2024 Oct;30(10):2202–4. doi: 10.3201/eid3010.240727 (PMC11431900; doi:10.3201/eid3010.240727)
Supplement: Appendix — Additional information about prosthetic valve endocarditis caused by Pasteurella dagmatis, Germany. [file 24-0727-Techapp-s1.pdf]

Article DOI: https://doi.org/10.3201/eid3010.240727

EID cannot ensure accessibility for supplementary materials supplied by authors. Readers who have difficulty accessing supplementary content should contact the authors for assistance.

Prosthetic Valve Endocarditis Caused by *Pasteurella dagmatis*, Germany

Appendix

Appendix Table. List of cases of infective endocarditis caused by *Pasteurella dagmatis**

Year	Country	Age/Sex	Animal contact	Complicating factors	Infected valve	Prosthetic valve	Definitive therapy	Surgery required	Authors
1972	United States (Vermont)	44/Male	Cat	Glomerulonephritis	Unknown (diastolic murmur)	No	penicillin G, penicillin V	No	Gump and Holden (*1*)
1994	United States (New Jersey)	55/Female	Cat	Vertebral osteomyelitis (L2/3)	Mitral valve	No	ceftriaxone	No	Sorbello et al. (*2*)
2001	United States (Florida)	78/Male	–	Vertebral osteomyelitis (L3/4), Glomerulonephritis	Aortic valve	Yes	ceftriaxone	No	Rosenbach et al. (*3*)
2012	Switzerland	77/Male	Cat	-	Aortic valve	Yes	penicillin G	Yes	Strahm et al. (*4*)
2024	Germany	81/Male	Cat	Paravalvular abscess	Aortic valve	Yes	penicillin G + levofloxacin	Yes	Rottmann et al. (this article)

*Four cases were previously reported in English language literature. Animal contact was a domestic cat in 4/5 cases. The aortic valve was most commonly infected. Of note, 2/5 cases presented with glomerulonephritis. All therapies included penicillin G or ceftriaxone and all patients survived the infection.

References

1. Gump DW, Holden RA. Endocarditis caused by a new species of *Pasteurella*. Ann Intern Med. 1972;76:275–8. PubMed https://doi.org/10.7326/0003-4819-76-2-275

2. Sorbello AF, O'Donnell J, Kaiser-Smith J, Fitzharris J, Shinkarow J, Doneson S. Infective endocarditis due to *Pasteurella dagmatis*: case report and review. Clin Infect Dis. 1994;18:336–8. PubMed https://doi.org/10.1093/clinids/18.3.336

3. Rosenbach KA, Poblete J, Larkin I. Prosthetic valve endocarditis caused by *Pasteurella dagmatis*. South Med J. 2001;94:1033–5. PubMed https://doi.org/10.1097/00007611-200194100-00020

4. Strahm C, Goldenberger D, Gutmann M, Kuhnert P, Graber P. Prosthetic valve endocarditis caused by a *Pasteurella dagmatis*-like isolate originating from a patient's cat. J Clin Microbiol. 2012;50:2818–9. PubMed https://doi.org/10.1128/JCM.00973-12
